# 3-Carb­oxy­phenyl­boronic acid–theophylline (1/1)

**DOI:** 10.1107/S1600536812029467

**Published:** 2012-07-04

**Authors:** Ventsi Dyulgerov, Rositsa P. Nikolova, Louiza T. Dimova, Boris L. Shivachev

**Affiliations:** a Bulgarian Academy of Sciences, Institute of Mineralogy and Crystallography, Acad G. Bonchev str. build. 107, 1113 Sofia, Bulgaria

## Abstract

The title two-component mol­ecular crystal [systematic name: 3-(dihy­droxy­boran­yl)benzoic acid–1,3-dimethyl-7*H*-purine-2,6-dione (1/1)], C_7_H_7_BO_4_·C_7_H_8_N_4_O_2_, comprises theophylline and 3-carb­oxy­phenyl­boronic acid mol­ecules in a 1:1 molar ratio. In the crystal, mol­ecules are self-assembled by O—H⋯O and N—H⋯N hydrogen bonds, generating layers parallel to (-209). The layers are stacked through π–π [centroid–centroid distance = 3.546 (2) Å] and C—H⋯π inter­actions.

## Related literature

For background to theophylline and boronic acids, see: Barnes (2003 ▶); Brittain (1999 ▶).
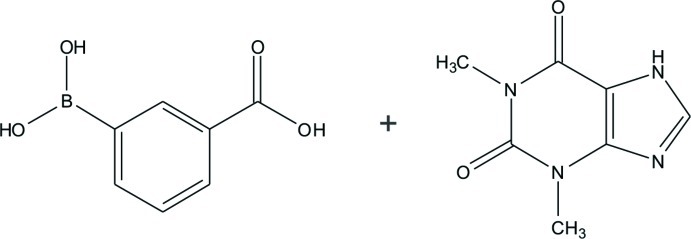


## Experimental

### 

#### Crystal data

C_7_H_7_BO_4_·C_7_H_8_N_4_O_2_
*M* *_r_* = 346.11
Monoclinic, 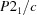

*a* = 13.185 (4) Å
*b* = 9.189 (3) Å
*c* = 13.287 (4) Å
β = 109.04 (3)°
*V* = 1521.7 (8) Å^3^
*Z* = 4
Mo *K*α radiation
μ = 0.12 mm^−1^
*T* = 290 K
0.32 × 0.3 × 0.28 mm

#### Data collection

Enraf–Nonius CAD-4 diffractometer
5890 measured reflections
2972 independent reflections
1949 reflections with *I* > 2σ(*I*)
*R*_int_ = 0.037
3 standard reflections every 120 min intensity decay: none

#### Refinement

*R*[*F* ^2^ > 2σ(*F* ^2^)] = 0.050
*wR*(*F* ^2^) = 0.145
*S* = 1.03
2972 reflections
231 parameters
H-atom parameters constrained
Δρ_max_ = 0.30 e Å^−3^
Δρ_min_ = −0.27 e Å^−3^

### 

Data collection: *CAD-4 EXPRESS* (Enraf–Nonius, 1994 ▶); cell refinement: *CAD-4 EXPRESS*; data reduction: *XCAD4* (Harms & Wocadlo, 1995 ▶); program(s) used to solve structure: *SHELXS97* (Sheldrick, 2008 ▶); program(s) used to refine structure: *SHELXL97* (Sheldrick, 2008 ▶); molecular graphics: *ORTEP-3 for Windows* (Farrugia, 1997 ▶); software used to prepare material for publication: *WinGX* (Farrugia, 1999 ▶).

## Supplementary Material

Crystal structure: contains datablock(s) global, I. DOI: 10.1107/S1600536812029467/kp2425sup1.cif

Structure factors: contains datablock(s) I. DOI: 10.1107/S1600536812029467/kp2425Isup2.hkl

Supplementary material file. DOI: 10.1107/S1600536812029467/kp2425Isup3.cml

Additional supplementary materials:  crystallographic information; 3D view; checkCIF report

## Figures and Tables

**Table 1:** Hydrogen-bond geometry (Å, °) *Cg* is the centroif of the C2–C7 ring.

*D*—H⋯*A*	*D*—H	H⋯*A*	*D*⋯*A*	*D*—H⋯*A*
O3—H3⋯O1^i^	0.82	1.94	2.753 (2)	168
O2—H2⋯O3^ii^	0.82	1.89	2.671 (2)	160
O4—H4*A*⋯O11^iii^	0.82	2.03	2.815 (3)	160
N2—H2*A*⋯N1^iv^	0.86	1.95	2.812 (3)	177
C14—H14*A*⋯*Cg*	0.96	2.59	3.483 (3)	155

Symmetry codes: (i) 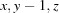; (ii) 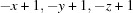; (iii) 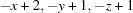; (iv) 
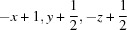
.

**Fig. 1.**
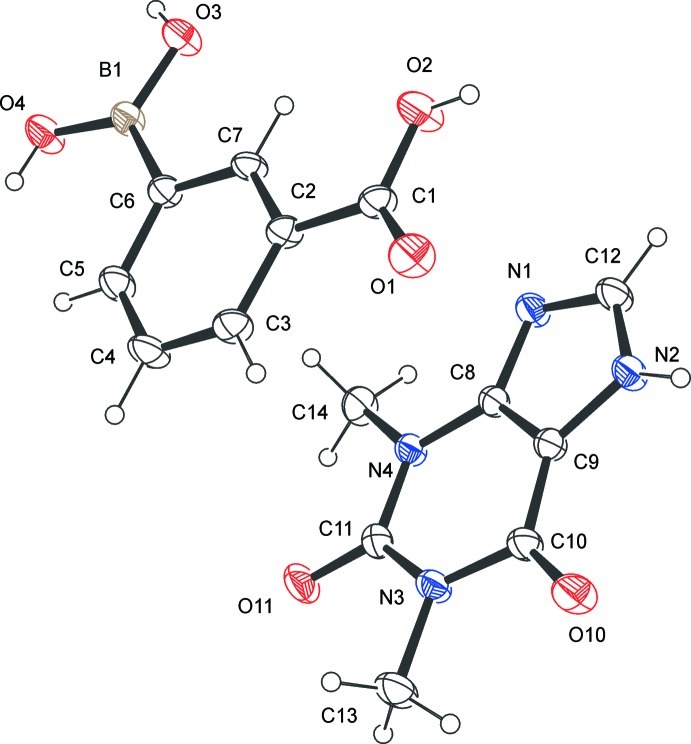
Molecular structure of the title compound, with atom-numbering scheme and 50% probability displacement ellipsoids.

**Fig. 2.**
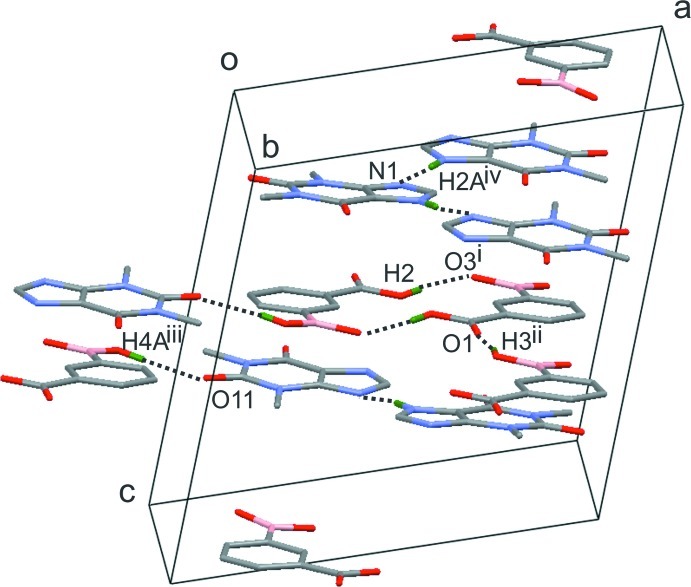
Crystal packing of the title compound. Hydrogen bonds are shown by dashed lines. Symmetry codes are used: (i) 1 - x, 1 - y, 1 - z; (ii)x, 1 + y, z; (iii) x - 1, y, z; (iv)x, y - 1, z.

**Fig. 3.**
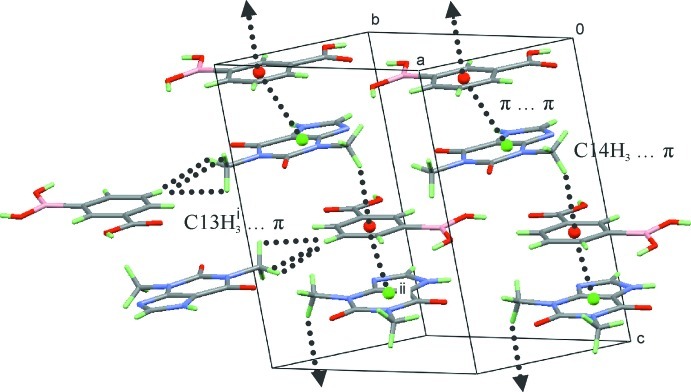
Projection of the structure showing the interactions (π–π and CH3···π) within the layers, denoted by dotted lines. Symmetry codes are: (i) 2 - x, 2 - y, 1 - z; (ii) x, 3/2 - y, 1/2 + z.

## supplementary crystallographic information

### Comment

Theophylline (1,3-dimethyl-1*H*-purine-2,6(3*H*,7H)-dione) is a xanthine derivative chemically similar to caffeine and theobromine (Barnes, 2003). Apart from new perspectives in organic chemistry, (*e.g.* Suzuki coupling reactions) boronic acids are emerging in the fields of crystal engineering, biochemistry and medicinal chemistry. In most cases the usage of the Active Pharmaceutical Ingredients (APIs) has been routinely restricted to salts, polymorphs, hydrates or solvates form (Brittain, 1999). Here we present the structure of the two-component molecular crystal theophylline (THEO) and 3-carboxyphenyl boronic acid (CPHB).

The ring systems are nearly planar with r.m.s. of 0.0045 and 0.0172 for the phenyl (C2/C3/C4/C5/C6/C7) and purine (C11/N4/C8/N1/C12/N2/C9/C10/N3) moieties. The boronic group is slightly twisted from the phenyl plane (the angle between the mean planes of the phenyl and B1/O3/O4 is 6.93 (8) °). The angle between the phenyl and purine mean planes of the two molecules is 4.52 (5) ° (Fig. 1). In the crystal structure neighbouring CPHB molecules form dimmers through O2—H2···O3 hydrogen bond and thus R2,2(16) motif is observed (Table 1). Adjacent dimmers are linked through O3—H3···O1 bond and thus a tetramer of CPHB is generated [*R*_4_,^4^(12) motif]. The O3—H3···O1 bond produces C1,1(8) chains that propagate along *b* axis. The THEO molecules are linked together by N2—H2A···N1 hydrogen bond producing C1,1(4) chain that propagate along *b* axis (Fig. 2). The CPHB and THEO molecules (chains) are linked *via* O4—H4···O11 hydrogen bond and form layers parallel to the plane (-2 0 9) (interlayer distance of 3.507 Å). In addition to the extensive hydrogen bonding the structure reveals weak CH_3_···π and π–π interactions (Fig. 3): (i) an almost parallel π-π stacking involving the PHB and THEO aromatic rings (offset of 1.04 Å and distance separation of 3.546 Å between C2/C3/C4/C5/C6/C7 and C8/C9/C10N3/C11/N4 ring centroids); (ii) an offset π–π interaction between C2/C3/C4/C5/C6/C7 and N1/C12/N2/C9/C10/N3/C11/N4/C8 rings (with distance separation and offset for afore mentioned centroids of 4.603 and 3.18 Å respectively); (iii) a CH_methyl_···..π interaction (C14···..CPHB ring, with C14 to C2/C3/C4/C5/C6/C7 centroid distance of 3.483 Å), and (iv) a T-shape CH_methyl_···π interaction between C13 and CPHB aromatic ring (with shortest distance, C13···C4 of 3.582 (4) Å) (Fig. 3).

### Experimental

Crystals of the title compound were obtained by slow evaporation of a 1:1 mol. mixture of theophylline and 3-carboxyphenyl boronic acid in water/MeOH (1:1 *v*/*v*) at room temperature.

### Refinement

All H atoms were placed in idealized positions (C—H_aromatic_ = 0.93 Å; *C*—H_methyl_ = 0.96 Å; N—H = 0.86 Å and O—H = 0.82 Å) and were constrained to ride on their parent atoms, with *U*_iso_(H) = 1.2*U*_eq_(C or N) and Uĩso~(H) = 1.5*U*_eq_(O)

### Crystal data

**Table:**

C_7_H_7_BO_4_·C_7_H_8_N_4_O_2_	*F*(000) = 720
*M**_r_* = 346.11	*D*_x_ = 1.511 Mg m^−^^3^
Monoclinic, *P*2_1_/*c*	Mo *K*α radiation, λ = 0.71073 Å
*a* = 13.185 (4) Å	Cell parameters from 22 reflections
*b* = 9.189 (3) Å	θ = 18.4–19.8°
*c* = 13.287 (4) Å	µ = 0.12 mm^−^^1^
β = 109.04 (3)°	*T* = 290 K
*V* = 1521.7 (8) Å^3^	Prism, colourless
*Z* = 4	0.32 × 0.3 × 0.28 mm

### Data collection

**Table:**

Enraf–Nonius CAD-4 diffractometer	*R*_int_ = 0.037
Radiation source: Enraf–Nonius FR590	θ_max_ = 26.0°, θ_min_ = 1.6°
Graphite monochromator	*h* = 0→16
non–profiled ω/2τ scans	*k* = −11→11
5890 measured reflections	*l* = −16→15
2972 independent reflections	3 standard reflections every 120 min
1949 reflections with *I* > 2σ(*I*)	intensity decay: none

### Refinement

**Table:**

Refinement on *F*^2^	Primary atom site location: structure-invariant direct methods
Least-squares matrix: full	Secondary atom site location: difference Fourier map
*R*[*F*^2^ > 2σ(*F*^2^)] = 0.050	Hydrogen site location: inferred from neighbouring sites
*wR*(*F*^2^) = 0.145	H-atom parameters constrained
*S* = 1.03	*w* = 1/[σ^2^(*F*_o_^2^) + (0.0783*P*)^2^ + 0.0279*P*] where *P* = (*F*_o_^2^ + 2*F*_c_^2^)/3
2972 reflections	(Δ/σ)_max_ < 0.001
231 parameters	Δρ_max_ = 0.30 e Å^−^^3^
0 restraints	Δρ_min_ = −0.27 e Å^−^^3^

### Special details

**Table:**

Geometry. All e.s.d.'s (except the e.s.d. in the dihedral angle between two l.s. planes) are estimated using the full covariance matrix. The cell e.s.d.'s are taken into account individually in the estimation of e.s.d.'s in distances, angles and torsion angles; correlations between e.s.d.'s in cell parameters are only used when they are defined by crystal symmetry. An approximate (isotropic) treatment of cell e.s.d.'s is used for estimating e.s.d.'s involving l.s. planes.
Refinement. Refinement of *F*^2^ against ALL reflections. The weighted *R*-factor *wR* and goodness of fit *S* are based on *F*^2^, conventional *R*-factors *R* are based on *F*, with *F* set to zero for negative *F*^2^. The threshold expression of *F*^2^ > σ(*F*^2^) is used only for calculating *R*-factors(gt) *etc*. and is not relevant to the choice of reflections for refinement. *R*-factors based on *F*^2^ are statistically about twice as large as those based on *F*, and *R*- factors based on ALL data will be even larger.

### Fractional atomic coordinates and isotropic or equivalent isotropic displacement parameters (Å^2^)

**Table:**

	*x*	*y*	*z*	*U*_iso_*/*U*_eq_	
C1	0.61757 (18)	0.7493 (2)	0.51103 (18)	0.0351 (5)	
C2	0.70843 (17)	0.6460 (2)	0.52797 (17)	0.0325 (5)	
C3	0.81135 (18)	0.7002 (2)	0.54821 (18)	0.0386 (6)	
H3A	0.8229	0.8001	0.5496	0.046*	
C4	0.89597 (19)	0.6057 (3)	0.5661 (2)	0.0448 (6)	
H4	0.965	0.6416	0.5788	0.054*	
C5	0.87869 (18)	0.4568 (2)	0.56527 (19)	0.0395 (6)	
H5	0.937	0.3944	0.5777	0.047*	
C6	0.77718 (18)	0.3978 (2)	0.54645 (18)	0.0335 (5)	
C7	0.69213 (17)	0.4961 (2)	0.52676 (17)	0.0322 (5)	
H7	0.6228	0.4606	0.5125	0.039*	
B1	0.7584 (2)	0.2280 (3)	0.5481 (2)	0.0366 (6)	
O1	0.62796 (14)	0.87975 (16)	0.51616 (15)	0.0515 (5)	
O2	0.52453 (13)	0.68396 (17)	0.49021 (17)	0.0531 (5)	
H2	0.4779	0.7446	0.4868	0.08*	
O3	0.65614 (12)	0.17673 (16)	0.51923 (15)	0.0450 (5)	
H3	0.6572	0.0876	0.522	0.067*	
O4	0.83877 (13)	0.12910 (17)	0.57757 (16)	0.0512 (5)	
H4A	0.8966	0.1715	0.5996	0.077*	
C8	0.66439 (16)	0.8169 (2)	0.27274 (17)	0.0308 (5)	
C9	0.65008 (17)	0.9642 (2)	0.27539 (18)	0.0328 (5)	
C10	0.73564 (17)	1.0648 (2)	0.29647 (19)	0.0356 (5)	
C11	0.85224 (17)	0.8449 (2)	0.31589 (18)	0.0353 (5)	
C12	0.49854 (19)	0.8503 (2)	0.2413 (2)	0.0423 (6)	
H12	0.4256	0.8334	0.2265	0.051*	
C13	0.93062 (19)	1.0874 (3)	0.3434 (2)	0.0505 (7)	
H13A	0.9747	1.0576	0.3021	0.076*	
H13B	0.9706	1.0788	0.4178	0.076*	
H13C	0.909	1.1868	0.3271	0.076*	
C14	0.7768 (2)	0.5983 (2)	0.2817 (2)	0.0427 (6)	
H14A	0.7807	0.5529	0.348	0.064*	
H14B	0.8414	0.5786	0.2659	0.064*	
H14C	0.7164	0.5602	0.2259	0.064*	
N1	0.57032 (15)	0.7444 (2)	0.25156 (16)	0.0385 (5)	
N2	0.54161 (14)	0.9822 (2)	0.25452 (16)	0.0393 (5)	
H2A	0.5082	1.0635	0.2507	0.047*	
N3	0.83506 (14)	0.9941 (2)	0.31754 (16)	0.0372 (5)	
N4	0.76421 (14)	0.75608 (19)	0.29013 (14)	0.0327 (4)	
O10	0.72989 (13)	1.19763 (17)	0.29774 (16)	0.0522 (5)	
O11	0.94299 (12)	0.79504 (18)	0.33659 (15)	0.0497 (5)	

### Atomic displacement parameters (Å^2^)

**Table:**

	*U*^11^	*U*^22^	*U*^33^	*U*^12^	*U*^13^	*U*^23^
C1	0.0373 (13)	0.0233 (11)	0.0456 (14)	−0.0044 (9)	0.0150 (10)	0.0005 (10)
C2	0.0348 (12)	0.0228 (11)	0.0406 (13)	−0.0039 (9)	0.0135 (10)	−0.0034 (9)
C3	0.0385 (13)	0.0252 (11)	0.0512 (15)	−0.0084 (10)	0.0134 (11)	−0.0032 (10)
C4	0.0294 (13)	0.0360 (13)	0.0674 (18)	−0.0112 (10)	0.0135 (12)	−0.0036 (12)
C5	0.0293 (12)	0.0322 (12)	0.0562 (15)	0.0027 (10)	0.0128 (11)	0.0011 (11)
C6	0.0314 (12)	0.0257 (11)	0.0430 (13)	−0.0013 (9)	0.0117 (10)	−0.0002 (9)
C7	0.0273 (11)	0.0234 (11)	0.0476 (14)	−0.0055 (9)	0.0145 (10)	−0.0010 (9)
B1	0.0303 (13)	0.0266 (13)	0.0516 (17)	0.0023 (10)	0.0117 (12)	0.0004 (11)
O1	0.0522 (11)	0.0198 (8)	0.0809 (13)	−0.0022 (7)	0.0195 (10)	−0.0018 (8)
O2	0.0342 (10)	0.0248 (8)	0.1026 (15)	0.0009 (7)	0.0254 (10)	−0.0011 (9)
O3	0.0310 (9)	0.0195 (8)	0.0829 (13)	0.0010 (6)	0.0164 (8)	0.0006 (8)
O4	0.0307 (9)	0.0272 (9)	0.0902 (14)	0.0027 (7)	0.0123 (9)	0.0057 (8)
C8	0.0260 (11)	0.0255 (11)	0.0398 (12)	0.0004 (9)	0.0091 (9)	0.0026 (9)
C9	0.0282 (11)	0.0248 (11)	0.0455 (13)	0.0022 (9)	0.0122 (10)	0.0031 (9)
C10	0.0294 (12)	0.0278 (12)	0.0494 (14)	−0.0012 (9)	0.0127 (10)	0.0017 (10)
C11	0.0285 (12)	0.0336 (12)	0.0412 (13)	0.0033 (10)	0.0080 (10)	0.0048 (10)
C12	0.0278 (12)	0.0291 (12)	0.0680 (16)	−0.0024 (10)	0.0128 (11)	0.0022 (11)
C13	0.0320 (13)	0.0371 (13)	0.0774 (19)	−0.0100 (10)	0.0112 (13)	0.0001 (12)
C14	0.0411 (14)	0.0251 (12)	0.0592 (16)	0.0055 (10)	0.0127 (12)	−0.0003 (10)
N1	0.0283 (10)	0.0258 (9)	0.0583 (13)	−0.0013 (8)	0.0101 (9)	0.0024 (9)
N2	0.0274 (10)	0.0228 (9)	0.0651 (13)	0.0017 (7)	0.0117 (9)	0.0022 (9)
N3	0.0254 (9)	0.0297 (10)	0.0535 (12)	−0.0035 (8)	0.0088 (8)	0.0009 (9)
N4	0.0269 (9)	0.0239 (9)	0.0465 (11)	0.0026 (7)	0.0106 (8)	0.0023 (8)
O10	0.0411 (10)	0.0232 (9)	0.0913 (14)	−0.0022 (7)	0.0203 (9)	−0.0017 (8)
O11	0.0258 (9)	0.0420 (10)	0.0761 (13)	0.0071 (7)	0.0097 (8)	0.0067 (8)

### Geometric parameters (Å, º)

**Table:**

C1—O1	1.206 (3)	C8—N4	1.378 (3)
C1—O2	1.312 (3)	C9—N2	1.375 (3)
C1—C2	1.487 (3)	C9—C10	1.414 (3)
C2—C3	1.387 (3)	C10—O10	1.223 (3)
C2—C7	1.393 (3)	C10—N3	1.407 (3)
C3—C4	1.372 (3)	C11—O11	1.226 (3)
C3—H3A	0.93	C11—N4	1.368 (3)
C4—C5	1.386 (3)	C11—N3	1.391 (3)
C4—H4	0.93	C12—N2	1.326 (3)
C5—C6	1.389 (3)	C12—N1	1.333 (3)
C5—H5	0.93	C12—H12	0.93
C6—C7	1.397 (3)	C13—N3	1.469 (3)
C6—B1	1.581 (3)	C13—H13A	0.96
C7—H7	0.93	C13—H13B	0.96
B1—O4	1.353 (3)	C13—H13C	0.96
B1—O3	1.360 (3)	C14—N4	1.468 (3)
O2—H2	0.82	C14—H14A	0.96
O3—H3	0.82	C14—H14B	0.96
O4—H4A	0.82	C14—H14C	0.96
C8—N1	1.354 (3)	N2—H2A	0.86
C8—C9	1.369 (3)		
			
O1—C1—O2	123.2 (2)	N2—C9—C10	132.2 (2)
O1—C1—C2	123.7 (2)	O10—C10—N3	121.1 (2)
O2—C1—C2	113.07 (18)	O10—C10—C9	127.3 (2)
C3—C2—C7	119.7 (2)	N3—C10—C9	111.63 (18)
C3—C2—C1	119.28 (19)	O11—C11—N4	121.3 (2)
C7—C2—C1	121.04 (19)	O11—C11—N3	121.1 (2)
C4—C3—C2	119.7 (2)	N4—C11—N3	117.57 (19)
C4—C3—H3A	120.2	N2—C12—N1	113.3 (2)
C2—C3—H3A	120.2	N2—C12—H12	123.4
C3—C4—C5	120.1 (2)	N1—C12—H12	123.4
C3—C4—H4	120	N3—C13—H13A	109.5
C5—C4—H4	120	N3—C13—H13B	109.5
C4—C5—C6	122.2 (2)	H13A—C13—H13B	109.5
C4—C5—H5	118.9	N3—C13—H13C	109.5
C6—C5—H5	118.9	H13A—C13—H13C	109.5
C5—C6—C7	116.63 (19)	H13B—C13—H13C	109.5
C5—C6—B1	122.0 (2)	N4—C14—H14A	109.5
C7—C6—B1	121.4 (2)	N4—C14—H14B	109.5
C2—C7—C6	121.7 (2)	H14A—C14—H14B	109.5
C2—C7—H7	119.1	N4—C14—H14C	109.5
C6—C7—H7	119.1	H14A—C14—H14C	109.5
O4—B1—O3	117.4 (2)	H14B—C14—H14C	109.5
O4—B1—C6	123.7 (2)	C12—N1—C8	103.52 (18)
O3—B1—C6	118.9 (2)	C12—N2—C9	106.80 (19)
C1—O2—H2	109.5	C12—N2—H2A	126.6
B1—O3—H3	109.5	C9—N2—H2A	126.6
B1—O4—H4A	109.5	C11—N3—C10	126.73 (18)
N1—C8—C9	111.54 (19)	C11—N3—C13	116.56 (18)
N1—C8—N4	126.55 (19)	C10—N3—C13	116.70 (18)
C9—C8—N4	121.91 (19)	C11—N4—C8	119.09 (18)
C8—C9—N2	104.89 (19)	C11—N4—C14	120.05 (18)
C8—C9—C10	122.96 (19)	C8—N4—C14	120.86 (18)
			
O1—C1—C2—C3	−1.7 (4)	C8—C9—C10—N3	−2.1 (3)
O2—C1—C2—C3	178.5 (2)	N2—C9—C10—N3	177.6 (2)
O1—C1—C2—C7	176.4 (2)	N2—C12—N1—C8	0.1 (3)
O2—C1—C2—C7	−3.3 (3)	C9—C8—N1—C12	0.0 (3)
C7—C2—C3—C4	0.5 (4)	N4—C8—N1—C12	−180.0 (2)
C1—C2—C3—C4	178.7 (2)	N1—C12—N2—C9	−0.2 (3)
C2—C3—C4—C5	−0.8 (4)	C8—C9—N2—C12	0.2 (3)
C3—C4—C5—C6	0.2 (4)	C10—C9—N2—C12	−179.6 (2)
C4—C5—C6—C7	0.8 (4)	O11—C11—N3—C10	−179.4 (2)
C4—C5—C6—B1	−178.6 (2)	N4—C11—N3—C10	0.8 (3)
C3—C2—C7—C6	0.6 (3)	O11—C11—N3—C13	1.0 (3)
C1—C2—C7—C6	−177.6 (2)	N4—C11—N3—C13	−178.8 (2)
C5—C6—C7—C2	−1.2 (3)	O10—C10—N3—C11	−178.2 (2)
B1—C6—C7—C2	178.3 (2)	C9—C10—N3—C11	1.9 (3)
C5—C6—B1—O4	6.6 (4)	O10—C10—N3—C13	1.4 (3)
C7—C6—B1—O4	−172.8 (2)	C9—C10—N3—C13	−178.5 (2)
C5—C6—B1—O3	−173.9 (2)	O11—C11—N4—C8	176.8 (2)
C7—C6—B1—O3	6.7 (4)	N3—C11—N4—C8	−3.4 (3)
N1—C8—C9—N2	−0.1 (3)	O11—C11—N4—C14	−3.0 (3)
N4—C8—C9—N2	179.9 (2)	N3—C11—N4—C14	176.9 (2)
N1—C8—C9—C10	179.7 (2)	N1—C8—N4—C11	−176.7 (2)
N4—C8—C9—C10	−0.3 (4)	C9—C8—N4—C11	3.2 (3)
C8—C9—C10—O10	178.0 (2)	N1—C8—N4—C14	3.0 (3)
N2—C9—C10—O10	−2.3 (4)	C9—C8—N4—C14	−177.0 (2)

### Hydrogen-bond geometry (Å, º)

Cg is the centroif of the C2–C7 ring.

**Table:**

*D*—H···*A*	*D*—H	H···*A*	*D*···*A*	*D*—H···*A*
O3—H3···O1^i^	0.82	1.94	2.753 (2)	168
O2—H2···O3^ii^	0.82	1.89	2.671 (2)	160
O4—H4*A*···O11^iii^	0.82	2.03	2.815 (3)	160
N2—H2*A*···N1^iv^	0.86	1.95	2.812 (3)	177
C14—H14*A*···*Cg*	0.96	2.59	3.483 (3)	155

Symmetry codes: (i) *x*, *y*−1, *z*; (ii) −*x*+1, −*y*+1, −*z*+1; (iii) −*x*+2, −*y*+1, −*z*+1; (iv) −*x*+1, *y*+1/2, −*z*+1/2.
